# Retraction Note: Solid lipid curcumin particles provide greater anti-amyloid, anti-inflammatory and neuroprotective effects than curcumin in the 5xFAD mouse model of Alzheimer’s disease

**DOI:** 10.1186/s12868-023-00785-5

**Published:** 2023-02-24

**Authors:** Panchanan Maiti, Leela Paladugu, Gary L. Dunbar

**Affiliations:** 1 Field Neurosciences Institute Laboratory for Restorative Neurology, Central Michigan University, 48859 Mt. Pleasant, MI USA; 2 Program in Neuroscience, Central Michigan University, 48859 Mt. Pleasant, MI USA; 3 Department of Psychology, Central Michigan University, 48859 Mt. Pleasant, MI USA; 4 Field Neurosciences Institute, St. Mary’s of Michigan, 48604 Saginaw, MI USA; 5 Department of Biology and Brain Research Laboratory, Saginaw Valley State University, 48604 Saginaw, MI USA

**Retraction Note**: ***BMC Neurosci*19, 7 (2018).**

10.1186/s12868-018-0406-3

The Editor has retracted this article at the corresponding author’s request. After publication, concerns were raised regarding similarities in the presented data. Specifically:

- In Fig. 6, the PFC 5xFAD + SLCP (2d) and CA1 5xFAD + Cur (2d) images appear highly similar;
- Also in Fig. 6, the CA3 5xFAD + Cur (2d) and 5xFAD + SLCP (2d) appear to originate from the same sample;
- Figure 8a GFAP 5xFAD (top) appears highly similar to Fig. 13a GFAP 5xFAD (bottom) with a brightness adjustment.

The authors checked their data and identified additional errors:

- In Fig. 11a, the two Iba-1 5xFAD images originated from the same sample;
- In Fig. 14a, the two Iba-1 5xFAD images originated from the same sample.

The Editor and the authors therefore no longer have confidence in the presented data.

All authors agree to this retraction.

[1] Maiti, P., Paladugu, L. & Dunbar, G.L. Solid lipid curcumin particles provide greater anti-amyloid, anti-inflammatory and neuroprotective effects than curcumin in the 5xFAD mouse model of Alzheimer’s disease. *BMC Neurosci***19**, 7 (2018). 10.1186/s12868-018-0406-3.

